# P08-10 Clustering of unhealthy lifestyle factors in occupational groups in the Swedish workforce

**DOI:** 10.1093/eurpub/ckac095.123

**Published:** 2022-08-29

**Authors:** Daniel Väisänen, Lena Kallings, Erik Hemmingsson, Elin Ekblom-Bak

**Affiliations:** Swedish school of sport and health sciences, Stockholm, Sweden

**Keywords:** risk score, occupational groups, unhealthy lifestyle factors

## Abstract

**Background:**

The physical activity pattern of the population, as well as the tasks of different occupational groups, have changed over the past decades. Hence, studies within and between different occupational groups, and not just between white and blue collar workers, are central for current risk group analyses. The aim was to study clustering of unhealthy lifestyle factors in different occupational groups in a large sample of men and women from the Swedish working population.

**Methods:**

72,855 individuals aged 18-75 years (41% women) from the Swedish working population who participated in a nationwide occupational health service screening between 2014-2019 were included in this cross-sectional descriptive study. Nine different occupational groups were identified based on the International Standard Classification of Occupation 2008. Exercise, diet, smoking habits and perceived health were self-reported. Cardiorespiratory fitness was estimated using a submaximal cycle test. Blood pressure and BMI was assessed through physical examination. Logistic regression modelling assessed OR (95%CI) for clustering of unhealthy lifestyle factors, defined as ‘3 of the following; low exercise, poor diet, daily smoking, poor perceived health, low fitness, high blood pressure and high BMI in the different occupational groups.

**Results:**

The OR (95% CI) for clustering of unhealthy lifestyle factors were, compared to managers that served as reference, 1.00 (0.89-1.11) for professionals, 1.25 (1.11-1.39) for associate professionals, 1.93 (1.71-2.18) for clerical support workers, 2.40 (2.14-2.70) for service and sales workers, 1.63 (1.29-2.05) for agricultural, forestry and fishery workers, 2.23 (1.99-2.49) for craft and related trades workers, 2.52 (2.25-2.83) for plant and machine operators, and assemblers, and 2.62 (2.26-3.05) for elementary occupations. Comparing occupational groups within ‘service and sales workers’ and ‘plant and machine operators, and assemblers’, revealed significantly higher OR for professionals in care workers (OR2.92 (2.55-3.34)) and in drivers (OR 3.32(2.86-3.87)) compared to each of the main occupational groups.

**Conclusion:**

There were large variations in clustering of unhealthy lifestyle-related factors between as well as within different white and blue collar occupations. This study suggest that targeted measures of health promotion are foremost needed in blue collar occupations, however with some white collar sub-occupations being at similar need as blue collar occupations.

